# Stress response regulators identified through genome-wide transcriptome analysis of the (p)ppGpp-dependent response in *Rhizobium etli*

**DOI:** 10.1186/gb-2011-12-2-r17

**Published:** 2011-02-16

**Authors:** Maarten Vercruysse, Maarten Fauvart, Ann Jans, Serge Beullens, Kristien Braeken, Lore Cloots, Kristof Engelen, Kathleen Marchal, Jan Michiels

**Affiliations:** 1 Centre of Microbial and Plant Genetics, Katholiek Universiteit Leuven, Kasteelpark Arenberg 20, 3001 Heverlee, Belgium

## Abstract

**Background:**

The alarmone (p)ppGpp mediates a global reprogramming of gene expression upon nutrient limitation and other stresses to cope with these unfavorable conditions. Synthesis of (p)ppGpp is, in most bacteria, controlled by RelA/SpoT (Rsh) proteins. The role of (p)ppGpp has been characterized primarily in *Escherichia coli *and several Gram-positive bacteria. Here, we report the first in-depth analysis of the (p)ppGpp-regulon in an α-proteobacterium using a high-resolution tiling array to better understand the pleiotropic stress phenotype of a *relA*/*rsh *mutant.

**Results:**

We compared gene expression of the *Rhizobium etli *wild type and *rsh *(previously *rel*) mutant during exponential and stationary phase, identifying numerous (p)ppGpp targets, including small non-coding RNAs. The majority of the 834 (p)ppGpp-dependent genes were detected during stationary phase. Unexpectedly, 223 genes were expressed (p)ppGpp-dependently during early exponential phase, indicating the hitherto unrecognized importance of (p)ppGpp during active growth. Furthermore, we identified two (p)ppGpp-dependent key regulators for survival during heat and oxidative stress and one regulator putatively involved in metabolic adaptation, namely extracytoplasmic function sigma factor EcfG2/PF00052, transcription factor CH00371, and serine protein kinase PrkA.

**Conclusions:**

The regulatory role of (p)ppGpp in *R. etli *stress adaptation is far-reaching in redirecting gene expression during all growth phases. Genome-wide transcriptome analysis of a strain deficient in a global regulator, and exhibiting a pleiotropic phenotype, enables the identification of more specific regulators that control genes associated with a subset of stress phenotypes. This work is an important step toward a full understanding of the regulatory network underlying stress responses in α-proteobacteria.

## Background

*Rhizobium etli *is a soil-dwelling α-proteobacterium that infects the roots of its leguminous host plant *Phaseolus vulgaris*, the common bean plant, in order to establish a nitrogen-fixing symbiosis [1-4]. Like most microorganisms in nature, *R. etli *primarily resides in a non-growing state in the soil, where it is confronted with diverse and stressful conditions, such as non-optimal temperatures and pH levels, near-starvation conditions and competition with other microbial populations [5]. Although growth is restricted, long periods of inactivity are sporadically interrupted by proliferation. This cycle of growth and starvation has been likened to a feast and famine lifestyle [6].

Sophisticated regulatory networks allow bacteria to sense and respond to a variety of environmental stresses to rapidly adjust their cellular physiology for survival. These networks comprise transcriptional regulators, sigma factors, proteases and small non-coding RNAs (ncRNAs) that interact in a complex manner in order to control the metabolic changes needed for adaptation [5]. The stringent response is a widespread global regulatory system, activated in response to various unfavorable growth conditions, and mediated by guanosine tetraphosphate (ppGpp) and guanosine pentaphosphate (pppGpp), collectively referred to as (p)ppGpp [7]. This alarmone coordinates entrance into the non-growing state by inducing a general reprogramming of gene regulation, thereby downregulating cellular processes needed for growth and upregulating processes needed for survival. As a result, the available resources are diverted from growth to allow adaptation of the cell to the non-growing state [8,9]. The central role of this alarmone in the general stress response during the stationary phase is also illustrated by the increased sensitivity of (p)ppGpp-deficient mutants in various species to diverse stress factors [10]. Therefore, studying the (p)ppGpp regulon may be useful to identify novel regulators involved in the stress adaptation.

In *Escherichia coli*, the stress-induced alarmone production depends on two enzymes: RelA and SpoT [7]. When amino acids are limiting, uncharged tRNAs that bind ribosomes stimulate the ribosome-associated RelA to synthesize (p)ppGpp. Subsequent recovery when conditions are favorable again requires degradation of the alarmone, which is catalyzed by SpoT. SpoT is a bifunctional enzyme that can also synthesize (p)ppGpp in response to carbon, iron, phosphorus and fatty acid scarcity. Having two (p)ppGpp synthetases/hydrolases appears to be an exclusive feature of the γ-subdivision of the proteobacteria, as Gram-positive bacteria and most other Gram-negative bacteria, including *R. etli*, possess only a single RelA/SpoT homolog - usually referred to as Rel or Rsh - that displays both activities [10]. Most Gram-positive species additionally encode small proteins that consist solely of a synthetase domain [11].

(p)ppGpp primarily regulates gene transcription [12,13]. Several models have been proposed to accommodate the effects of (p)ppGpp on transcription. One of these models, the affinity model, argues for an increase in the availability of free RNA polymerase (RNAP) with increasing (p)ppGpp levels. As this alarmone binds near the active site of RNAP, the stability of the ribosomal RNA (*rrn*) open complexes decreases. Consequently, (p)ppGpp will induce promoters with low RNAP affinity, such as cell maintenance and stress response genes [14,15]. In another model, the σ factor competition model, the binding affinity of alternative sigma factors increases with increasing (p)ppGpp-levels compared to the housekeeping sigma factor σ^70^. This results in a decrease of σ^70^-bound RNAP and a downregulation of growth-related promoters that are dependent on high concentrations of σ^70^-bound RNAP for maximal expression [10,12,16]. In addition to regulating sigma factor activity, (p)ppGpp is also required for sigma factor expression, as is the case for the stationary phase sigma factor σ^S^, the heat shock sigma factor σ^H ^and the sigma factor controlling nitrogen metabolism, σ^54^, in *E. coli *[17,18]. Hence, these models for gene regulation of (p)ppGpp should be considered as working in concert. Finally, the recently identified cofactor DksA was demonstrated to stabilize binding of RNAP to (p)ppGpp, resulting in enhanced repression or stimulation of transcription in *E. coli*. However, the interaction between (p)ppGpp and DksA appears to be more complex as both factors also have independent and opposing effects on gene expression in *E. coli *[13,19,20].

In agreement with (p)ppGpp's central role in stress adaptation, the alarmone was shown to be crucial in many complex physiological processes such as biofilm formation by *Listeria monocytogenes*, *E. coli *and *Streptococcus mutans*, development of multicellular fruiting bodies in *Myxococcus xanthus *and development of competence in *Bacillus subtilis *[10]. In addition, a fast growing number of reports demonstrate (p)ppGpp to be important during host interactions in diverse pathogens such as *Vibrio cholerae*, *Pseudomonas aeruginosa*, *Legionella pneumophila*, *Francisella novicida*, *Enterococcus faecalis *and *Streptococcus pneumoniae *[21-24]. Furthermore, various transcriptome studies showed that the alarmone (p)ppGpp is situated high up in the hierarchy of interconnected regulators in *E. coli*, controlling the expression and/or function of many other regulators such as Lrp, the cAMP receptor protein CRP, the integration host factor IHF, the flagellar master regulator FlhDC, the redox status sensing regulator ArcA and the morphogene BolA [6,8,18,25,26].

(p)ppGpp also affects key aspects of the symbiosis between rhizobia and their leguminous host plants. In *Sinorhizobium meliloti*, a *rsh *mutant is defective in nodulation of *Medicago sativa *and overproduces the exopolysaccharide succinoglycan, which is crucial for root infection [27]. In *R. etli*, (p)ppGpp controls the physiological adaptation of the bacterium to the endosymbiotic state [28,29]. Although the *rsh *mutant induces nodulation, the bacteroids are morphologically different compared to the wild type, and nitrogen fixation activity is drastically reduced. Several nitrogen fixation and quorum-sensing genes, essential for symbiosis, were shown to be part of the alarmone regulon, including the symbiotic σ^N ^that is required for expression of nitrogen fixation genes [29]. Recently, a detailed phenotypic analysis of the *rsh *(previously referred to as *relA *or *rel*_Ret_) mutant showed a prominent role for the alarmone in the general stress response of *R. etli *during free-living growth and symbiosis [30].

In order to obtain new insights into the molecular basis of adaptation of *R. etli *to unfavorable growth conditions, we performed a genome-wide transcriptome analysis to compare global gene expression between the wild type and a *rsh *mutant during different free-living growth phases.

This study is the first in-depth analysis of (p)ppGpp-dependent gene regulation in an α-proteobacterium, revealing notable differences from the well-studied role of (p)ppGpp in *E. coli*. Of the many detected (p)ppGpp targets that may contribute to the observed stress phenotypes of the *rsh *mutant, we performed a phenotypic analysis of three specific previously uncharacterized regulators, that is, sigma factor EcfG2/PF00052, DNA-binding transcription factor CH00371 and serine kinase PrkA/CH02817. Our results show that the stress phenotypes of mutants lacking EcfG2 or CH00371 correspond to a subset of the *rsh *mutant phenotypes, while PrkA may be involved in metabolic adaptation. In addition, we identified several upstream and downstream elements in the stress response pathways of these three novel (p)ppGpp-dependent regulators, providing added detail to the complex picture of the role of (p)ppGpp in *R. etli*.

## Results and Discussion

### Experimental design of the transcriptome analysis

Previously, we reported on the crucial role of (p)ppGpp during symbiosis and free-living growth in *R. etli *CNPAF512 using a *rsh *mutant [29,30]. Based on these findings, we decided to carry out a transcriptome analysis to characterize to what extent (p)ppGpp deficiency affects gene expression in *R. etli*. The intracellular (p)ppGpp content of the *R. etli *wild type, *rsh *mutant and complemented *rsh *mutant was determined previously [29], showing the *rsh *mutant to be (p)ppGpp-deficient. However, due to the sensitivity of the assay, the presence of trace amounts of (p)ppGpp in the *rsh *mutant, possibly resulting from the presence of an as yet unidentified synthetase gene, cannot be ruled out.

At the time of the experimental setup, only the genomic DNA sequence of *R. etli *CFN42 was available [31]. Therefore, a custom whole-genome microarray for *R. etli *CFN42 as well as a CFN42-derived *rsh *mutant was constructed. Phenotypic analysis of this mutant showed that a lack of (p)ppGpp results in an extended lag phase in different media, an altered morphology and a 75% reduction of nitrogen fixation activity in plants inoculated with the CFN42 *rsh *mutant compared to the wild type (data not shown). All phenotypes could be fully complemented by providing *rsh *of CNPAF512 *in trans *and are in agreement with our previously published *rsh *mutant analyses [29,30].

To determine the role exerted by the alarmone (p)ppGpp in the regulation of transcription during free-living growth and growth arrest of *R. etli*, total RNA samples were taken at three different time points corresponding to early and late exponential and stationary phase, respectively (Additional file 1).

### Global overview of gene expression

**Figure 1:**
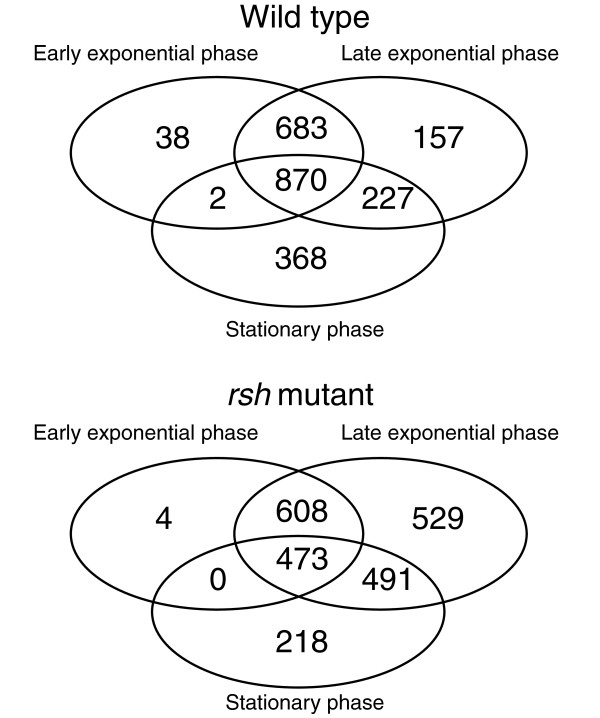
**Detectable gene expression overview**. The number of genes expressed above the detection threshold in each condition and the overlap between the different conditions are shown in Venn diagrams. Upper and lower diagrams represent expression in the wild type and *rsh *mutant, respectively.

The *R. etli *CFN42 genome contains 6,030 annotated protein-encoding genes, 67 pseudo genes, 3 rRNA operons and 50 tRNA genes. Recently, we described an additional 89 ncRNA genes [32]. In both the wild type and *rsh *mutant, over 97% (or (683 + 870)/1,593) of protein-encoding genes that are transcribed above the detection limit (see Materials and methods) during early exponential growth are also expressed during late exponential growth. In addition, numerous genes are induced in the course of growth, as 20% (or (157 + 227)/1,937) of the genes expressed in late exponential phase are not transcribed during early growth (Figure 1).

**Figure 2:**
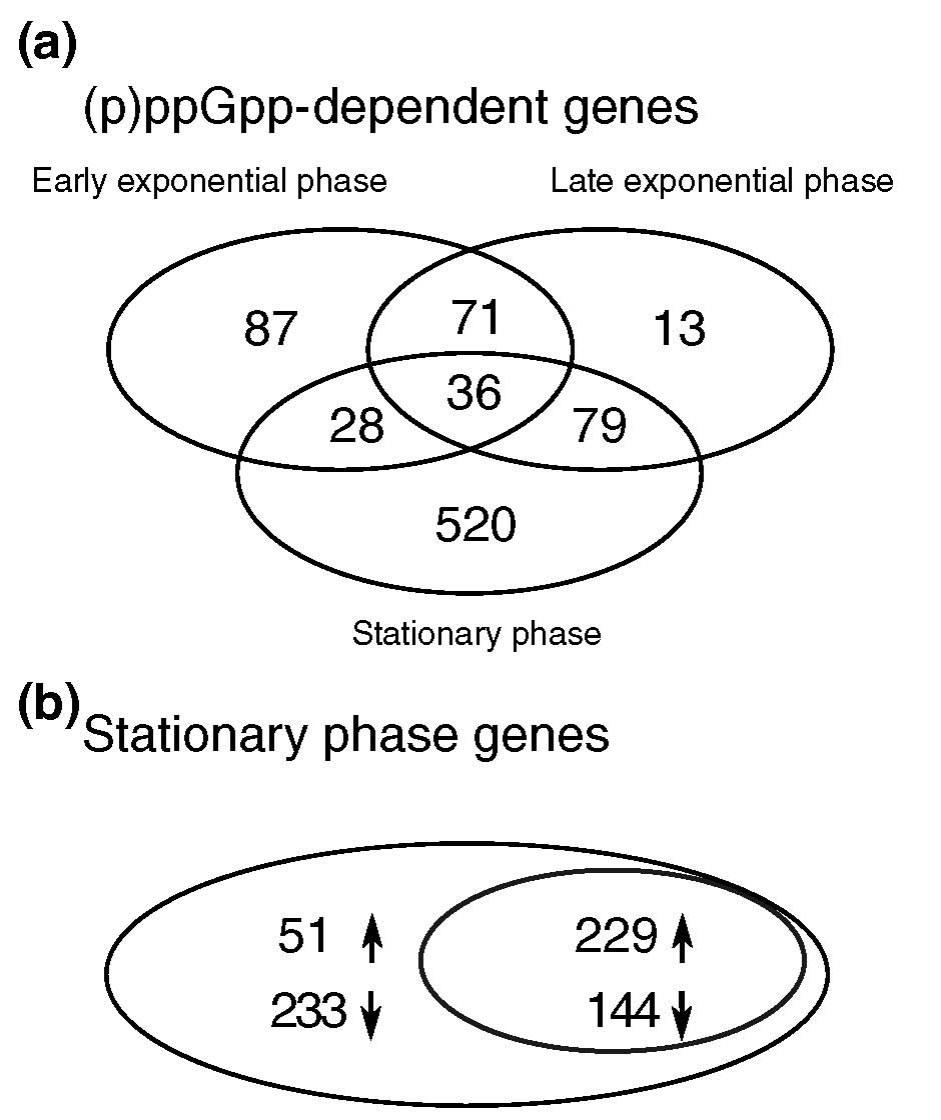
**(p)ppGpp-dependent gene expression**. **(a) **Venn diagram of all differentially expressed (p)ppGpp-dependent genes during early exponential phase, late exponential phase and stationary phase. **(b) **Venn diagram of all genes expressed during stationary phase (large ellipse). The overlap with (p)ppGpp-dependent genes (see (a)) shows all (p)ppGpp-dependent stationary phase genes (small ellipse). Upwards and downwards oriented arrows indicate gene induction and repression, respectively.

We identified a large number of differentially expressed genes during exponential and stationary phase, both (p)ppGpp-dependent and independent, the former being consistent with the role of *rsh *as a global regulator described in other species [33-35]. The extent of differential expression is illustrated by the ratio/intensity MA plots in Additional file 2. Alarmone dependency was determined by comparing gene expression of the wild type and *rsh *mutant during each of the three sampled growth phases (Figure 2a). A total of 834 (p)ppGpp-dependent genes with an expression ratio of at least two-fold were found. Approximately half of these genes (520) were expressed exclusively during stationary phase and only a minority (36) were found to be (p)ppGpp-dependent during all growth conditions.

By comparing expression in the wild type during stationary and early exponential phase, we identified 657 stationary phase genes (Figure 2b), representing 11% (or 657/6,030) of the annotated protein-coding genes. The overlap of (p)ppGpp-dependent genes and stationary phase genes shows that just over half (57% or (229 + 144)/657) of stationary phase genes are (p)ppGpp-dependent. Because 61% (or 229/373) of these were upregulated (Figure 2b), the alarmone (p)ppGpp seems to have a primarily inducing role in *R. etli*. A comparable number of (p)ppGpp-dependent genes were found in other bacteria: 490 (11%) of all genes in *E. coli *and 194 (6%) in *Corynebacterium glutamicum *after (p)ppGpp-induction by serine hydroxymate [18,34], 589 genes (7%) upon induction of (p)ppGpp synthesis in *Streptomyces coelicolor *[35] and 373 (18%) of all genes after treatment with mupirocin in *Streptococcus pneumoniae *[23].

**Figure 3:**
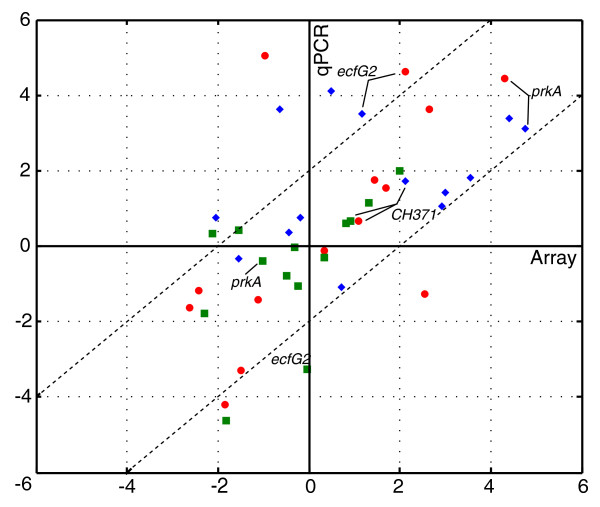
**RT-qPCR validation of the microarray data**. Expression of 14 genes was determined using RT-qPCR for the wild type and *rsh *mutant in early exponential phase and stationary phase. The log_2_-transformed mean values of two biological replicates were used to report three different fold changes for each gene (Y-axis) compared to the respective microarray fold changes (X-axis). See Additional file 6 for a complete list of the plotted fold change values. Red dots, wild type stationary phase versus early exponential phase. Blue diamonds, wild type versus *rsh *mutant in stationary phase. Green squares, wild type versus *rsh *mutant in exponential phase. The fold changes for *ecfG2*/PF00052, CH00371 and *prkA*/CH02817 are indicated.

The microarray data were confirmed by analyzing the expression levels of 14 representative genes using reverse transcription-quantitative PCR (RT-qPCR; see Materials and methods). For each gene, expression during early exponential phase and stationary phase in the wild type and *rsh *mutant was measured so that three different ratios could be plotted versus the respective ratios obtained by microarray analysis (Figure 3), showing the array data to be in good agreement with the RT-qPCR data.

### The effect of (p)ppGpp on global gene expression during stationary phase

**Figure 4:**
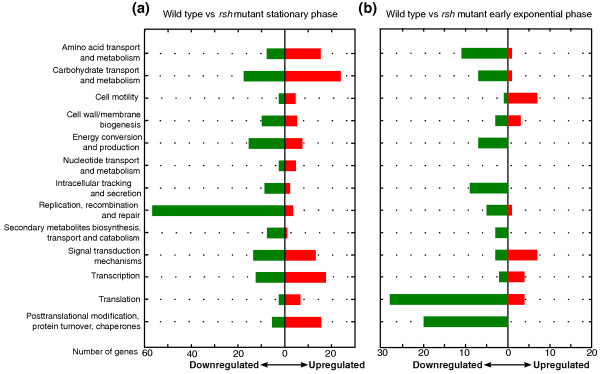
**Differentially expressed genes grouped by functional categories**. Up- and downregulated (wild type versus *rsh *mutant) genes are indicated by red and green bars, respectively, representing the number of genes per functional category. Functional categories of the RhizoBase database were used [92]. **(a) **Stationary phase data of wild type versus *rsh *mutant. **(b) **Early exponential data of wild type versus *rsh *mutant.

During stationary phase in *E. coli*, the alarmone (p)ppGpp induces a downregulation of processes involved in cell growth, such as DNA replication and translation, and an upregulation of specific metabolic pathways to cope with certain nutrient deficiencies as well as general stress responses to protect the cell against immediate and future harmful conditions. In order to better understand the role of (p)ppGpp in the global reprogramming of *R. etli*'s transcriptome, we compared the expression of wild type and *rsh *mutant during stationary phase. As samples were taken approximately 6 hours after growth arrest, the observed differences in expression include both direct and indirect effects caused by a lack of alarmone. Of the 663 differentially expressed genes, 292 and 371 were upregulated and downregulated, respectively, in the wild type compared to the *rsh *mutant (Figure 2a). These genes were further grouped based on predicted functional role and category (Additional file 3). An overview of the functional categories (Figure 4a) shows the mutant to be less well adapted to the non-growing lifestyle as more growth-associated genes, involved in cell wall biosynthesis, energy production and intracellular trafficking and secretion, are induced in the mutant. Notably, the replication and recombination category is strongly represented in the mutant due to the high number of insertion sequence (IS)-related genes that show expression. An equal number of genes with unknown function were up- and downregulated. In the following paragraphs, selected functional categories, primarily focused on regulation and possible links with the pleiotropic stress phenotype, will be discussed in more detail.

#### Transcriptional regulators and signal transduction

The link between changes in extracellular conditions and concomitant adaptation of genome expression involves a combination of sensors, transporters, phosphorylation cascades and the modulation of transcription factors [36]. Most of these belong to the 'transcription' and 'signal transduction' categories, of which 29 and 26 genes are differentially expressed, respectively, in the wild type compared to the (p)ppGpp-deficient mutant at onset of growth arrest (Additional file 3).

**Figure 5:**
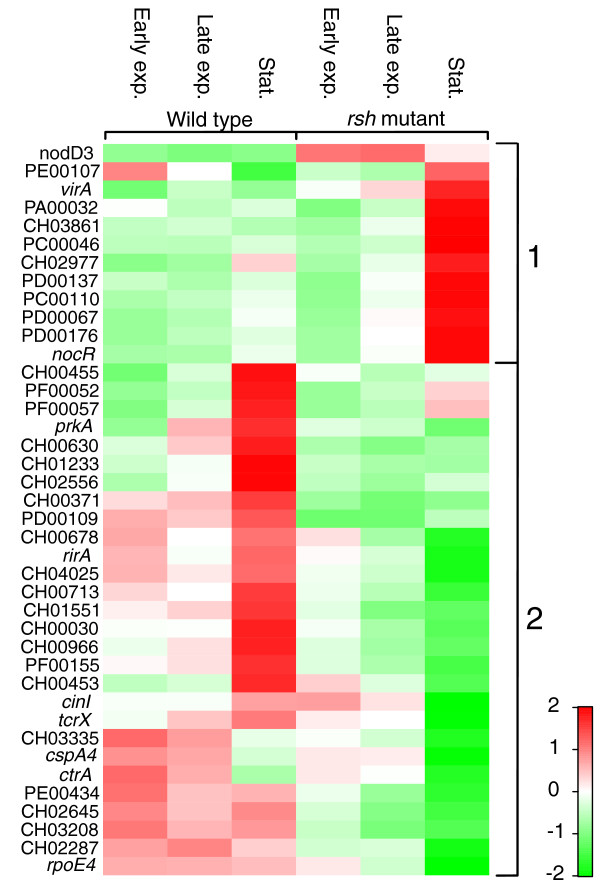
**Clustering of differentially expressed signal transduction and transcription-related genes**. The heat map visualizes the expression profiles of all differentially expressed genes belonging to the transcription category and signal transduction category in the wild type and *rsh *mutant during stationary phase. The expression values in each row were standardized by subtraction of the mean and division by the standard deviation and hierarchically clustered. Expression values are reflected by red-green coloring as indicated. Genes showing similar expression patterns are grouped as follows: group 1, genes under negative (p)ppGpp control during stationary phase; group 2, genes under positive (p)ppGpp control during stationary phase. Exp., exponential; Stat., stationary.

By clustering the differentially expressed genes of these two categories, we identified two main groups (Figure 5). The first group contains genes that are under negative (p)ppGpp control during primarily the stationary phase and include the LysR transcriptional regulators *nocR *and *nodD3*, the two-component sensor kinase *virA *and two diguanylate cyclases, PD00137 and PE00107. The second group contains genes that are under positive (p)ppGpp control during primarily the stationary phase, encoding among others the transcriptional regulators RirA and BolA-like CH02287, the CarD-like regulator CH04025, the two-component response regulators CH02556 and CH03335, and the *N*-acyl-L-homoserine lactone (AHL) synthase CinI.

Several of these transcriptional regulators have previously been shown to play a role in the adaptation to adverse conditions in other species and can partly explain the pleiotropic stress phenotype of the *rsh *mutant. In *E. coli*, BolA controls expression of a number of cell wall proteins, is partially responsible for the coccoid morphology of stationary phase cells and is also expressed in a (p)ppGpp-dependent manner, being under control of RpoS [5,18,33]. Reduced BolA levels may therefore contribute to the altered morphology of the *R. etli rsh *mutant. Furthermore, expression of the global iron-responsive regulator RirA that controls the synthesis of heme, FeS-clusters and bacterioferritin in rhizobia, is under positive (p)ppGpp control as well [37,38]. Accordingly, expression of bacterioferritin (*bfr*) was positively upregulated in the *R. etli *wild type, suggesting that (p)ppGpp contributes to iron homeostasis. Conversely, a lack of iron may cause an increase in the level of (p)ppGpp in order to regulate iron homeostasis in the cell as reported in *E. coli *and *B. subtilis *[39,40]. Since iron plays a crucial role in the oxidative stress response, incomplete iron sequestration may also contribute to the increased oxidative stress sensitivity of the *rsh *mutant [30,41]. Other regulators under positive (p)ppGpp control include two members of the cold shock protein family (CspA3, CspA4), a putative member of the UspA family (CH01233), the SOS response regulator LexA and the two-component regulator TcrX. TcrX is orthologous to PhyR of *Methylobacterium extorquens*, which regulates many stress response genes and was shown to play a role in the osmotic stress response in *R. etli *as well [42,43].

#### Sigma factors

*R. etli *CFN42 possesses 23 sigma factors that determine the promoter specificity of the RNAP holoenzyme by binding to the core enzyme. Therefore, differential expression and/or activity of sigma factors can redirect global gene expression. During exponential growth, transcription is largely under control of the housekeeping sigma factor σ^70 ^as its binding affinity for RNAP and intracellular concentration are much higher compared to the other sigma factors. These alternative sigma factors have specific regulons and will redirect transcription upon unfavorable conditions. Bacteria like *R. etli *that have a complex lifestyle or encounter diverse environmental conditions usually display an increased number of sigma factors [5,44].

Upon transition to stationary phase, the reversible switch to a less σ^70^-dominated expression in *E. coli *is accomplished not solely by (p)ppGpp but also by DksA and the anti-σ^70 ^factor Rsd. In *R. etli*, expression of *dksA *is reduced over eight-fold in stationary phase compared to early exponential phase in a (p)ppGpp-independent manner. The role of DksA in α-proteobacteria is so far unknown. Furthermore, no Rsd homolog is found in *R. etli *or other other α-proteobacteria. *R. etli *may compensate for the lack of a specific anti-σ^70 ^factor, as we observed a (p)ppGpp-independent drop in expression of the housekeeping sigma factor *sigA *to below the detection limit while expression in *E. coli *of σ^70 ^remains constant during stationary phase.

Of all the alternative sigma factors, only the extracytoplasmic function (ECF) sigma factor PF00052 was upregulated at least two-fold during stationary phase compared to early exponential phase in the wild type. The ECF sigma factor *rpoE4 *is expressed at the same level during all conditions in the wild type, but dropped below the expression threshold during stationary phase in the *rsh *mutant. Consequently, two ECF sigma factors, PF00052 and *rpoE4*, were upregulated over two-fold in the wild type compared to the *rsh *mutant during stationary phase (Figure 5). In *E. coli*, only the level of the stationary phase sigma factor *rpoS *(σ^S^) increases with (p)ppGpp concentration and plays a crucial role as global regulator in the (p)ppGpp-dependent stress response [45,46]. In contrast, ε- and α-proteobacteria, including *R. etli*, lack such a stationary phase sigma factor and, so far, it is unclear which system takes over this function.

Our data suggest that both PF00052 and RpoE4 may be important sigma factors in *R. etli *adaptation to stationary phase and possibly fulfill a role similar to RpoS in *E. coli*. First, both sigma factors are expressed during stationary phase. Second, both sigma factors are the most highly upregulated (p)ppGpp-dependent alternative sigma factors during stationary phase. Third, both share considerable sequence similarity and were recently classified in a group of proposed general stress response sigma factors that is exclusively found in α-proteobacteria [47]. Fourth, it was recently shown in *R. etli *that RpoE4 regulates gene expression in response to several stress conditions including oxidative, saline and osmotic stress. Fifth, we found that PF00052 is also involved in the (p)ppGpp-dependent stress response and is in part functionally redundant with RpoE4 (see below).

Transcriptome analysis of an *R. etli rpoE4 *mutant and overexpression strain revealed 98 genes to be regulated by this sigma factor [42]. Since transcription of *rpoE4 *is (p)ppGpp-dependent, we investigated to what extent the reported RpoE4 regulon is (p)ppGpp-dependent. In total, 60 of the 98 genes belonging to the reported regulon are differentially expressed in our data (Additional file 4). Of these genes, 82% are (p)ppGpp-dependent and 92% are up- or downregulated during stationary phase compared to early exponential phase in the wild type. Upon *rpoE4 *overexpression, 74% of the reported upregulated genes were found to be (p)ppGpp-dependent.

Considering the RpoE4-regulated genes, all 16 genes predicted to encode proteins associated with cell envelope biogenesis are also (p)ppGpp-dependent. Similarly, *E. coli*'s sole ECF sigma factor, σ^E^, regulates many genes involved in the biogenesis and stress response of the cell envelope [48,49]. Other RpoE4 and (p)ppGpp-dependent genes include a putative Mn-catalase (CH00462), a putative pyridoxine-phosphate oxidase (CH03474), an alpha-glucoside ABC transporter (*algE*), and a CarD-like transcriptional regulator (CH04025). The latter is a crucial regulator in *Mycobacterium tuberculosis *that is upregulated in response to oxidative stress, DNA damage and starvation [48,49]. The above suggests that the pleiotropic stress phenotype of the *R. etli rsh *mutant can be explained, at least in part, by downregulation of (p)ppGpp-dependent sigma factors that play a crucial role in orchestrating the stress response.

#### Non-coding RNAs

Our data indicate that (p)ppGpp controls expression of many protein-coding genes. In addition, we identified 33 alarmone-dependent ncRNAs expressed during stationary phase. Of these, 28 were positively regulated by (p)ppGpp, including one glycine riboswitch, 17 novel ncRNAs, 4 previously identified but uncharacterized ncRNAs, and the 6 well characterized ncRNAs (6S RNA, tmRNA, signal recognition particle 4.5S RNA, RNase P, and ctRNA of plasmids p42d and p42e) (Additional file 5). Only five ncRNAs, all novel, were negatively regulated by (p)ppGpp.

So far, no ncRNAs have been reported to be (p)ppGpp-dependent in any organism. However, in recent years, an increasing number of ncRNAs have been found to be regulated by alternative sigma factors in *E. coli*, *Salmonella enterica *serovar Typhimurium, *L. monocytogenes*, *B. subtilis *and *S. coelicolor *[50-53]. Therefore, the (p)ppGpp-dependent ncRNAs of *R. etli *could be regulated by alternative sigma factors as well. Additionally, ncRNAs can also regulate sigma factors, as is the case for σ^S ^in *E. coli *whose translation is regulated by DsrA and RprA [50-53].

The level of 6S RNA was almost 14-fold lower in the alarmone-deficient mutant during stationary phase in *R. etli*. This is unlike in *E. coli*, where 6S RNA is not under (p)ppGpp control either *in vitro *or *in vivo *[54,55]. However, 6S RNA transcription appears to be complexly regulated in *E. coli *as several stress regulators, such as Fis, H-NS, Lrp and StpA, were shown to be inhibitors under *in vitro *conditions [55]. Recently, transcriptional analysis of a 6S RNA-deficient mutant showed 273 genes to be differentially expressed during stationary phase. Surprisingly, loss of 6S RNA in *E. coli *also resulted in an increase of the basal (p)ppGpp level mediated by an altered activity of SpoT and not RelA [56]. Therefore, 6S RNA is clearly embedded in stationary phase adaptation, although its association with (p)ppGpp in *R. etli *and *E. coli *may differ.

Expression of bacterial RNase P and tmRNA was almost 13- and 6-fold downregulated, respectively, in the *R. etli rsh *mutant compared to the wild type. Although the synthesis and processing of tRNA is expected to be downregulated during growth arrest, 38% of the tRNAs were upregulated in the wild type during stationary phase compared to early exponential phase and 56% of the tRNAs were upregulated in an alarmone-dependent manner. The upregulation of bacterial RNase P during stationary phase in an alarmone-dependent manner is in line with the unexpected upregulation of several tRNAs as RNase P is required to process the 5' end of precursor tRNAs. Expression of tmRNA is also upregulated in *R. etli*. This alarmone-dependence of tmRNA expression has not been reported in *E. coli*, although a lack of 6S RNA results in a three-fold higher expression of the SmpB protein, which acts together with tmRNA [56]. However, the expression level of tmRNA was not reported. Interestingly, the 6S RNA mutation is compensated for by an increase of the basal (p)ppGpp level, which indicates that the tmRNA/SmpB system might be alarmone-dependent in *E. coli *also. Still, (p)ppGpp is not needed for mRNA cleavage in the A site of the ribosome by tmRNA [57]. In contrast, both tmRNA and *smpB *of *Streptococcus pyogenes *were shown to be upregulated in a *relA*-independent amino acid starvation response [58].

#### Translational apparatus

In addition to inducing general stress and nutrient scavenging regulons, the accumulation of (p)ppGpp upon growth arrest in *E. coli *is characterized by a stringent downregulation of expression of the translational apparatus as a mechanism to fine-tune the metabolically expensive process of protein synthesis according to the growth state of the cell [9,59]. As expected, during the stationary phase all 56 genes encoding ribosomal proteins were downregulated in the *R. etli *wild type compared to the exponential phase. However, nearly all (53 out of 56) of these were downregulated in the *rsh *mutant as well. Although this (p)ppGpp-independent downregulation is in conflict with the established *E. coli *paradigm of the stringent response, a similar response was described in a *rel *mutant of *Corynebacterium glutamicum *upon addition of serine hydroxamate [9,34,59]. Therefore, the difference in transcriptional regulation of ribosomal protein expression during growth arrest suggests that the stringent response in *R. etli *may deviate from the classical model in *E. coli*.

Other genes encoding parts of the translational machinery that were positively regulated by (p)ppGpp in *R. etli *include the homolog of *E. coli yhbH *(CH00406) and two EF-Tu elongation factors (*tufA*, *tufB*). In *E. coli*, YhbH is involved in the temporary storage or dimerization of ribosomes during stationary phase. This process was shown to contribute to the survival of *E. coli *[28]. In accordance with our data, the YhbH ortholog of *B. subtilis *(*yvyD*) is also under positive (p)ppGpp control [60]. However, in contrast to the positive (p)ppGpp-dependent regulation of *tufA *and *tufB *in *R. etli*, TE-Tu factors in *E. coli *and *B. subtilis *were previously shown to be under negative control of (p)ppGpp [8,60]. Interestingly, translation factors such as TE-Tu are GTPases that can bind (p)ppGpp and associate with the ribosome, indicating that they may have a downstream role in (p)ppGpp-dependent gene regulation [13].

#### Post-translational modification, repair and recombination

The (p)ppGpp-dependent stress adaptation during stationary phase involves 20 genes belonging to the post-translational modification category, of which 15 were positively regulated. These include several components of the ATP-dependent Clp protease system, such as *clpX*, *clpP2*, *clpP3*, *clpA *and *clpS*, as well as the ATP-dependent proteases *lon *and *ftsH *[61]. These proteases allow cells to cope with misfolded or denatured proteins, the abundance of which increases during stress conditions, such as heat stress, in order to prevent protein aggregation and to enable recycling of amino acids [5]. A similar (p)ppGpp-dependent regulation was observed for *clpA *in *E. coli *as well as *clpP1 *and *clpC *in *C. glutamicum *[33,34]. Thus, the (p)ppGpp-dependent increase of tmRNA in *R. etli *correlates with the increase in proteases as the Clp system and Lon are needed to degrade tmRNA-tagged polypeptides in *E. coli *[62].

Proteases and chaperones are also involved in regulating transcriptional regulators and other growth-phase regulated proteins, such as RpoS, Dps and GlnA in *E. coli *[63]. Therefore, by controlling proteolysis, the alarmone (p)ppGpp mediates the cellular reprogramming of *R. etli *at the post-transcriptional level as well. Other positively controlled genes include the probable serine protease CH01273, the small heat shock protein PF00472 as well as genes required to cope with oxidative stress, such as *osmC *and *grlA*.

Rather unexpectedly, very few genes of the repair and recombination category were under positive stringent control. However, several IS-related genes were negatively controlled by (p)ppGpp, including 47 transposases, one resolvase, and one integrase. Therefore, these data suggest that the alarmone may assist in repressing insertional activity and mobility of IS-related elements.

#### Other processes

The impact of (p)ppGpp as a global regulator of transcription is further illustrated by its control of genes involved in diverse cellular processes. In *E. coli*, the alarmone plays a central role in restructuring metabolism upon nutrient starvation and growth arrest, thereby increasing the range of active metabolic pathways and nutrient scavenging potential [33]. In *R. etli*, the alarmone likely has a similar role in metabolism as differential gene expression was detected for 22 genes involved in amino acid metabolism, 41 genes in carbohydrate metabolism, 9 in lipid metabolism and 22 in energy production.

(p)ppGpp was shown in *E. coli *to induce amino acid biosynthesis pathways depending on the availability of limiting amino acids. However, compared to exponential phase, no clear upregulation during stationary phase of one or more specific amino acid pathways was found in the *R. etli *wild type. Only a few genes involved in amino acid metabolism were positively controlled by (p)ppGpp (*phhA*, *cysE1*, *glnA2*, *trpE*). In addition, 13 amino acid synthesis genes (*trpF*, *trpA*, *hisB*, *asnB*, *aroQ1*, *aroF*, *ilvI*, *aatA*, *lysC*, *argG2*, *tyrA*, *leuD*, *asd*) along with the P-II regulator *glnB*, which regulates glutamine synthetase in response to nitrogen levels, were downregulated in a (p)ppGpp-independent manner during stationary phase compared to exponential phase. Therefore, during stationary phase, amino acid biosynthesis in *R. etli *is downregulated rather than upregulated as in *E. coli*.

Several genes encoding key enzymes of carbohydrate metabolism were induced by (p)ppGpp, including the transaldolase *tal *of the pentose pathway, *glgC *involved in starch and sucrose metabolism, the glycolytic gene *fbaB *and the gene encoding trehalose-6-phophatase, *otsB*. These genes were also shown to be under positive control by (p)ppGpp in *E. coli *upon amino acid starvation [33]. FbaB is a fructose-bisphosphate aldolase whose reaction product can exert feedback control on the glycolytic flux and is also required for ribosome recycling during carbon starvation [6]. Moreover, OtsB produces the disaccharide trehalose from trehalose-6-phosphate, which is produced by OtsA using UDP-glucose and glucose-6-phosphate. Not only is trehalose an energy and carbon source, it also stabilizes and protects proteins and membranes from dehydration, oxidation and cold [64]. Recently, it was shown that all three trehalose synthesis pathways known to date are present in *S. meliloti*. However, only the OtsA pathway is important for osmo-inducible trehalose synthesis [65]. In *R. etli*, overexpressing *otsA *improves symbiotic efficiency and drought tolerance of its host *P. vulgaris *[66]. During stationary phase, *otsA *and *otsB *have a different expression pattern; *otsB *is induced by (p)ppGpp while *otsA *is constitutively expressed in the wild type but under negative (p)ppGpp control upon growth arrest. It is possible that this (p)ppGpp-dependent regulation of trehalose synthesis contributes to the previously observed increased sensitivity of the *rsh *mutant to osmotic stress [30].

The link between the stringent response and the available carbon sources remains unclear. In *E. coli*, the (p)ppGpp synthetase/hydrolase SpoT interacts with acyl carrier proteins (ACPs) of fatty acid metabolism [67]. *R. etli *contains four acyl carrier proteins, of which only two (*acpP*, *acpXL*) were expressed during growth and downregulated upon growth arrest independently of (p)ppGpp. In contrast to *E. coli*, no clear (p)ppGpp-dependent regulation of lipid metabolism genes was observed. Also, most of the nucleotide biosynthesis genes are downregulated during stationary phase compared to exponential phase in the wild type, reflecting the decreased need for nucleotides. Only six nucleotide biosynthesis genes were found to be under control of (p)ppGpp in *R. etli*. This is in accordance with the observed (p)ppGpp-independent downregulation of ribosomal proteins.

As well as regulating *R. etli*'s biosynthetic potential, (p)ppGpp also controls its transport capacity during stationary phase. Twenty-one genes related to ABC transporters were under positive (p)ppGpp control, such as *potF*, *dppA*, *proX*, *aglK*, and *gguB*, while 12 were repressed. Most of these transporters allow for the uptake of amino acids, peptides and monosaccharides. In addition, two secretion-associated genes (*secB *and *pilA*) were upregulated and seven genes involved in type IV secretion were downregulated (*virB1a*, *2a*, *D4*, *B6a*, *B8a*, *B8d*, *B10*). Interestingly, the pilin subunit *pilA *was the most highly expressed protein-encoding gene in *R. etli *during stationary phase.

Energy production drops during the stationary phase as more than 25 genes predicted to be involved in oxidative phosphorylation were downregulated compared to exponential phase in the wild type. In contrast to *E. coli*, 76% of the differentially expressed genes that belong to the energy production category, 95 in total, are not under (p)ppGpp control [33]. During free-living growth, *R. etli *uses cytochrome aa_3 _terminal oxidases, encoded by *ctaCDGE*, *coxPONM *and CH00981-CH00985 [31,68]. The *ctaCDGE *terminal oxidase was downregulated during stationary phase in both the wild type and the *rsh *mutant. On the other hand, the *coxPONM *alternative terminal oxidase was upregulated during stationary phase in the wild type but not in the *rsh *mutant. The third probable terminal oxidase was not expressed. Therefore, the alternative terminal oxidase *coxPONM *is likely to play an important role during (p)ppGpp-dependent stationary phase adaptation.

In addition to the decrease in energy production, flagellum synthesis and motility is also downregulated during stationary phase, reflecting that it is a highly energy demanding process. In *E. coli*, flagellar genes are under positive (p)ppGpp control [18,19,69]. Similarly in *R. etli*, (p)ppGpp positively regulates flagellar gene expression. However, this regulation occurs primarily during the exponential phase instead of the stationary phase, as 25 of the 35 flagellar genes were expressed above threshold during the exponential phase compared to 10 during the stationary phase. Of the latter, three flagellar hook-related genes (*flgD*, *flgE*, *flgL*) and two flagellin synthesis regulators (*flaF*, *flbT*) were upregulated in the wild type compared to the *rsh *mutant [70]. FlgD forms a scaffold on which the hook subunit FlgE polymerizes on the envelope-embedded rod to form the flexible hook structure. FlgL is a junction protein connecting the rigid flagellar filament. In short, (p)ppGpp regulates several crucial flagellar genes in *R. etli*.

### The effect of (p)ppGpp on global gene expression during early exponential phase

By comparing the expression data of the wild type and *rsh *mutant during early exponential growth, we identified 203 differentially expressed genes, of which 59 were under positive stringent control and 144 under negative stringent control. This is surprising as transcription during the exponential phase of a (p)ppGpp-deficient mutant and the wild type is generally thought to be very similar. The alarmone is considered to be a stationary phase or growth arrest-specific messenger that switches the cellular metabolism to a non-growing state. During favorable growth conditions, (p)ppGpp is produced at a low basal level and rapidly accumulates in response to growth-perturbing conditions. Furthermore, during the exponential phase, a (p)ppGpp-deficient mutant of *E. coli *is phenotypically very similar to the wild type, although a decreased growth phase-independent thermotolerance has been reported [71]. However, to the best of our knowledge, to this date no detailed comparison of global transcription during exponential growth has been described for a wild type and *relA spoT *mutant of *E. coli*, which serves as the stringent response model organism. Still, it was recently shown that almost 300 genes were differentially expressed in an *rpoS *mutant of *E. coli *during exponential growth, even though RpoS is known as the stationary phase sigma factor [72]. This would suggest that a difference in expression during logarithmic growth in a (p)ppGpp-deficient mutant could be expected as (p)ppGpp regulates the expression and activity of RpoS in *E. coli*. Moreover, a major difference in expression during growth in the absence of (p)ppGpp was also previously observed in *M. tuberculosis *and *C. glutamicum *[34,73]. Our data are in agreement with these reports, showing that the low basal level of (p)ppGpp is functionally relevant during active growth. This additional function is also in agreement with the observed increase in sensitivity to several acute and chronic stresses of a *R. etli rsh *mutant during exponential growth [30].

**Figure 6:**
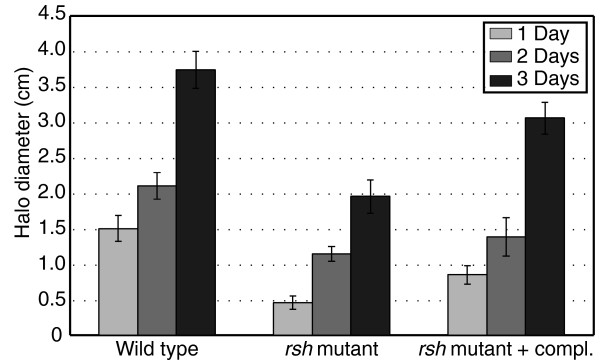
**Swimming motility test**. Swimming halo diameter observed on 0.2% agar TY plates on three consecutive days for wild type, *rsh *mutant and complemented *rsh *mutant. The mean values and standard deviation of five biological replicates are shown. The differences over time are statistically significant between the different strains (P < 0.001).

A comparison of the (p)ppGpp-dependent genes during early exponential phase and stationary phase showed that only 50 genes were differentially expressed in both states. Of this fraction, only half of the genes showed similar positive or negative control during both phases. This suggests that the function of (p)ppGpp differs during active growth and growth arrest, possibly through involvement of other regulators. To further understand the impact and role of the alarmone during exponential growth, we again grouped the up- and downregulated genes in functional categories (Figure 4b). As 71% of these genes were under negative (p)ppGpp regulation, the alarmone plays a primarily repressing role during logarithmic growth, in contrast to the observed predominantly inducing role upon growth arrest. For example, the alarmone induces 19 transporters during stationary phase while it represses 12 during early exponential phase. Other genes under negative (p)ppGpp control include 10 conjugal transfer proteins, 5 IS-related transposases and 26 ribosomal proteins. In contrast, nine motility genes were upregulated by (p)ppGpp, such as three of the four basal-body rod proteins (*flgBCG*), one of the three flagellar switch proteins that interact with the chemotaxis system (*fliN*) and three chemotaxis proteins (*motA*, *cheW5*, *cheY1*). Therefore, (p)ppGpp has a similarly inducing role on flagellar genes during growth as observed upon growth arrest. A swimming test on 0.2% agar plates corroborates this observation (Figure 6). The (p)ppGpp-deficient mutant showed reduced swimming activity compared to the wild type, a phenotype that could be partially complemented by providing the *rsh *gene *in trans*. Hence, the alarmone is required for optimal motility, as was also previously reported for *E. coli *[19,69].

Remarkably, in addition to growth-related genes, many post-translational modification genes were under negative stringent control in the *rsh *mutant. These comprise numerous chaperones, including the three major ones, *tig*, *dnaK *and *groEL-groES*, involved in folding of new proteins as well as in proper assembly of unfolded proteins and refolding of misfolded proteins generated under stress conditions [74]. DnaK is also involved in chromosomal DNA replication and is part of the osmotic stress response, in addition to *osmC *[74]. Other upregulated heat shock proteins in the *rsh *mutant include four peptidases (*hslV*, *lon*, *traF*, *htpX2*) and one protease (*ftsH*). Although exponentially growing cells are considered to be less stressed, this increased expression of many heat shock proteins in actively growing cells in the absence of (p)ppGpp might indicate a defect or disruption in protein homeostasis, rather then merely an increase in translational activity. Therefore, this stress response during growth is in accordance with increased stress sensitivity of the *rsh *mutant as observed previously [30].

### Functional analysis of three (p)ppGpp-dependent regulators

To gain insight into the (p)ppGpp-controlled adaptation of *R. etli *to the stationary phase and diverse stresses as previously reported [30], we selected three different types of previously uncharacterized regulators based on their strongly (p)ppGpp-dependent expression during stationary phase and belonging to the group of transcriptional regulators (*ecfG2*, *phrR*) or signal transduction (*prkA*). The microarray expression patterns of these regulators were confirmed using RT-qPCR (Figure 3). A phenotypical analysis was performed on the corresponding knockout strains to determine the regulators' contribution to the (p)ppGpp-regulated stress response.

#### Extracytoplasmic function sigma factor PF00052 or EcfG2

**Figure 7:**
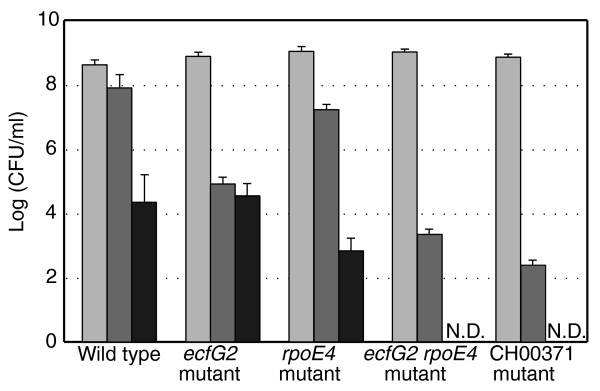
**Stress survival of (p)ppGpp-dependent regulator mutants**. Survival of the wild type, *ecfG2 *mutant, *rpoE4 *mutant, *ecfG2*-*rpoE4 *mutant and CH00371 mutant was determined by plating on TY medium after stress treatment and is shown as the mean log(colony forming units (CFU)/ml) of three biological replicates with error bars corresponding to standard deviations. Light gray bars represent control samples incubated at 30°C for the same time period as test samples. Dark gray bars represent samples incubated for 30 minutes in the presence of 10 mM H_2_O_2 _at 30°C. Black bars represent samples incubated for 30 minutes at 45°C. N.D., no colonies detected.

*R. etli *PF00052 is the most highly upregulated alternative sigma factor during stationary phase in the wild type and upregulated over two-fold compared to the *rsh *mutant. Analysis of the PF00052 knockout mutant showed decreased survival following oxidative stress, approximately three orders of magnitude lower compared to the wild type (Figure 7). In accordance, the *rsh *mutant also displays an increased oxidative stress phenotype [30]. In contrast to the *rsh *mutant, however, the PF00052 mutant does not show a decreased viability after osmotic or heat shock.

Recently, a comprehensive phylogenetic analysis classified the ECF sigma factor family into 43 groups supported by domain architecture, genomic context conservation and potential targets [47]. *R. etli *contains 18 ECF sigma factors, of which three (*rpoE4*, *sigK *and PE00004), in addition to PF00052, were expressed during growth arrest as well. RpoE4 and PF00052 are 42% identical and both belong to theECF15/EcfG group, which exclusively contains α-proteobacterial ECFs, such as EcfG1 of *Methylobacterium extorquens*, RpoE2 of *S. meliloti *and SigT of *C. crescentus*. This group of proposed general stress response sigma factors is characterized by a conserved genomic context that encodes an EcfG-like sigma factor, a cytoplasmic NepR-like anti-sigma factor, a PhyR-like response regulator and a sensor histidine kinase. When the latter perceives a signal, it phosphorylates the regulator, which in turn binds to the anti-sigma factor, thereby releasing the sigma factor and initiating a signal transduction cascade [47]. In case of *R. etli rpoE4*, an anti-sigma factor (CH03274) and a sensor/regulator (*tcrXY *or CH03275-CH03276) are found upstream of *rpoE4*, and *tcrX *is transcribed in a strongly (p)ppGpp-dependent way (Additional file 3). Though PF00052 is also a member of the EcfG group, a similar genomic context was not recognized. Several other α-proteobacteria also have two EcfG representatives, one that is present in the conserved genomic context while the other is not - for example, *S. meliloti *1021 and *Agrobacterium tumefaciens *str. C58 [47]. A revised ECF nomenclature has been proposed [47] and has recently been adopted for EcfG-like ECF sigma factors in *M. extorquens *[43] and *Bradyrhizobium japonicum *[75]. Accordingly, we will hereafter refer to PF00052 as EcfG2.

Due to the high similarity between RpoE4 and EcfG2, we also analyzed the stress sensitivity of an *rpoE4 *mutant. This revealed a decrease in survival of one order of magnitude upon oxidative and heat stress compared to the wild type (Figure 7). Hence, the *rpoE4 *mutant exhibits an oxidative stress phenotype less severe than the *ecfG2 *mutant but, in contrast to the latter, a significant heat stress phenotype. This suggests that RpoE4 and EcfG2 control expression of, at least in part, non-overlapping sets of target genes. To determine if both sigma factors function completely independently in these stress responses, an *rpoE4*-*ecfG2 *double mutant was constructed. After heat or oxidative stress treatment, the double mutant showed higher sensitivity than either of the single mutants separately (Figure 7). Moreover, survival of the double mutant is even lower than expected based upon the respective phenotypes of the single mutants, indicating a synergistic effect. Hence, we conclude that both sigma factors have partially overlapping functions in the (p)ppGpp-mediated stress response of *R. etli *to the tested stress conditions.

*R. etli *has two heat shock sigma factors that may contribute to the observed heat stress phenotype. RpoH1 was shown to be the main heat shock sigma factor, though a more complete response requires RpoH2 [76]. The promoter region of both sigma factors contains an EcfG-like binding site.

Recently, expression of *R. etli rpoH2 *was shown to be under positive control of RpoE4, while expression of *rpoH1 *is not [42]. To determine whether either or both *rpoH *sigma factors are regulated by EcfG2, their expression was analyzed by qPCR. The expression level of *rpoH1 *was not altered in the *ecfG2 *mutant compared to the wild type, while the transcription level of *rpoH2 *was reduced by approximately 25%. In the *rpoE4*-*ecfG2 *double mutant, expression of *rpoH2 *was reduced over 100-fold, confirming its RpoE4-dependency (data not shown). In contrast, the level of *rpoH1 *was upregulated over 2.5-fold in the double mutant. This increase is more likely a way to compensate for the impaired heat shock response, rather than a negative control of its expression.

Partial redundancy of EcfG-like sigma factors may occur in other α-proteobacteria as well. In *S. meliloti*, two EcfG-like proteins are encoded by *rpoE2 *and *rpoE5*. RpoE2 was previously reported to regulate many stress response genes during stationary phase. In agreement with our observations, an *S. meliloti rpoE2 *mutant showed increased sensitivity to H_2_O_2 _during stationary phase and *rpoE5 *is upregulated under heat stress [77,78].

#### Putative transcriptional regulator CH00371 or PhrR

CH00371 encodes a putative DNA-binding transcriptional regulator of unknown function belonging to the xenobiotic response element family. Expression of this gene is under positive (p)ppGpp-control during all growth phases, although most pronounced upon growth arrest. CH00371 is 80% identical to PhrR, a putative repressor protein of *S. meliloti*. This regulator was shown to be induced by low pH, hence designated PhrR for pH-regulated [79]. In order to investigate whether CH00371 plays a role in the acid stress response, growth of the *R. etli *wild type and CH00371 mutant was examined at pH levels ranging from pH 3.5 to 10. No growth difference was observed, either at acidic or basic pH (data not shown). In addition to low pH, oxidative stress agents and heat shock at neutral pH induce *phrR *in *S. meliloti *as well. Therefore, survival of the CH00371 mutant was determined under oxidative stress and after heat shock. Compared to the wild type, survival decreased by over four orders of magnitude upon oxidative shock following exposure to hydrogen peroxide (Figure 7). No survival was observed after heat treatment. Moreover, a plate assay demonstrated growth inhibition of a CH00371 mutant on medium containing H_2_O_2 _but not in the presence of the superoxide generators menadione and paraquat, nor of the organic hydroperoxide producer cumene hydroperoxide (data not shown).

In order to identify downstream elements in the regulatory cascade mediated by CH00371 during *R. etli *growth arrest, we carried out qPCR expression analysis of a selection of genes presumed to be associated with the oxidative and heat stress responses (Additional file 6). These candidate target genes were selected based on a literature search and sequence analysis. Several of these genes were downregulated. *recA*, a key regulator of the SOS response involved in DNA repair, and *osmC*, an osmotically induced peroxidase, were 70% and 30% downregulated in the CH00371 mutant compared to the wild type, respectively. The superoxide dismutase *sodC *was downregulated by 20%. Surprisingly, expression of *katG *was not significantly altered despite the oxidative stress phenotype. However, the expression level of *katG *was very low. This may indicate that KatG primarily exerts its function upon induction by oxidative shock. In addition, oxidative homeostasis is likely impaired in the CH00371 mutant as 14 genes related to oxidative stress resistance, such as *gshB*, *sufBCD *and *cysK*, showed increased expression of over 25% compared to the wild type. Interestingly, four genes of the EcfG2-RpoE4-regulon (CH00600, CH01778, CH01802 and CH02172) were also downregulated over 25%, possibly contributing to the observed heat stress phenotype.

Furthermore, the expression level of CH00371 was not altered in the *ecfG2 *mutant and *ecfG2 rpoE4 *double mutant compared to the wild type, nor vice versa (data not shown). Hence, these novel regulators exert their role in the observed stress phenotypes of the respective mutants independently of each other.

#### Putative serine kinase CH02817 or PrkA

**Figure 8:**
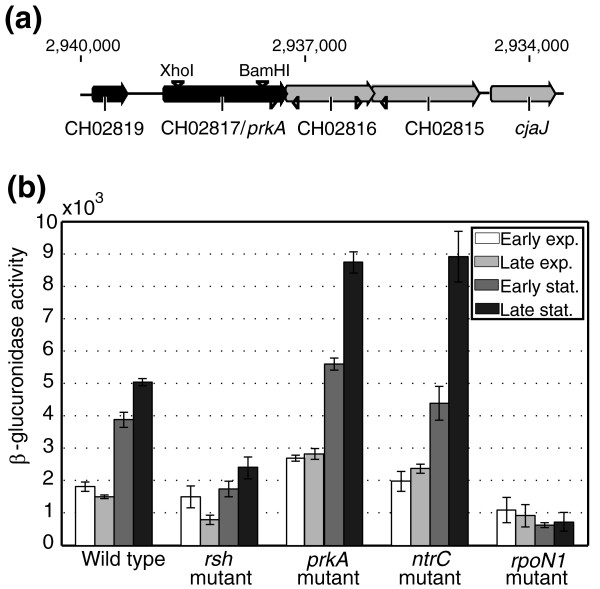
***prkA *genomic context and expression analysis**. **(a) ***R. etli prkA *is the first gene of a three-gene operon. Open reading frames are represented by right facing arrows, genomic coordinates are indicated above. Restriction sites for the deletion insertion of the *prkA *mutant are depicted by downwards facing triangles, and primer sites for RT-PCR used to determine *prkA *operon structure are depicted by left and right facing triangles. **(b) **Expression of *prkA*-*gusA *transcriptional reporter fusion was monitored in different *R. etli *mutant backgrounds during growth in AMS succinate. The strains were the wild type *R. etli *CFN42, *rsh *mutant, *prkA *mutant, *ntrC *mutant and *rpoN1 *mutant. Expression levels are shown in Miller units and are the means of three biological replicates with error bars representing the standard deviation. Exp., exponential; Stat., stationary.

CH02817 or *prkA *is upregulated over 27-fold in the wild type compared to the *rsh *mutant during stationary phase, making it the most strongly (p)ppGpp-induced gene detected in our array. This was confirmed by RT-qPCR (Figure 3). PrkA belongs to the PrkA family of serine protein kinases and is highly conserved, with homologs in many eubacteria and archae suggesting a conserved function. *E. coli *and *B. subtilis *PrkAs were shown to phosphorylate serine residues of proteins [80,81] and display 66% and 34% identity with the *R. etli *ortholog, respectively. Protein phosphorylation usually changes the function of the target by modulating its activity, its localization or interaction with other proteins, thereby converting extracellular signals into cellular responses, such as adaptation of the central metabolism, production of secondary metabolites and pathogenicity [82]. Although the specific regulatory function of PrkA remains unknown, the *B. subtilis *ortholog was shown to be an important inner spore coat protein under control of the developmental sigma factor σ^E ^[83]. Furthermore, *prkA *is part of a highly conserved gene cluster together with the two downstream genes CH02816 and CH02815. This suggests that *prkA *is likely the first gene of a three-gene operon in *R. etli *(Figure 8a), which we confirmed by RT-PCR (data not shown). The second operon gene encodes a protein containing a von Willebrand Factor type A domain and the third encodes a SpoVR-like protein. So far, these genes have not been functionally characterized.

To determine the function of *prkA *in *R. etli*, we constructed a non-polar deletion mutant. A phenotypical analysis of this mutant, including survival during stationary phase, osmotic stress, oxidative stress and heat stress, revealed no clear stress phenotype. Therefore, PrkA does not seem to play a crucial role in the stress response of *R. etli*. This is rather unexpected given the high level of (p)ppGpp-dependency and strong induction under stress conditions in other organisms. In *E. coli *and *S*. Typhimurium, the *prkA *homolog, annotated as *yeaG*, was also shown to be highly upregulated by (p)ppGpp upon entry into stationary phase as part of the RpoS regulon [81]. In addition, *yeaG *is upregulated by Lrp during stationary phase in *E. coli *[45,46]. Other stress conditions were also reported to induce *yeaG *expression, such as acid and osmotic stress in *E. coli *and sublethal concentrations of polymyxin in *S*. Typhimurium [6].

Additionally, PrkA was postulated to be involved in nitrogen metabolism or the nitrogen starvation response in *E. coli *based on its potential association with NtrCB and GlnP [46,84]. To examine the transcriptional regulation of *prkA *in *R. etli*, we monitored expression of a transcriptional *prkA*-*gusA *promoter fusion in various genetic backgrounds (Figure 8b). β-Glucuronidase activity was measured during exponential and stationary phase under the same conditions used for microarray sampling, confirming that the expression of *prkA *is highly upregulated upon growth arrest in the wild type and positively controlled by (p)ppGpp. Moreover, increased expression of *prkA *was observed in a *prkA *mutant, indicating that *prkA *is negatively autoregulated. Expression of *prkA *was also shown to be regulated by NtrC and RpoN1. NtrC is a transcriptional regulator involved in nitrogen assimilation and growth in nitrogen-limited conditions, as well as a member of the σ^N^-dependent activator family [85]. RpoN1 codes for the main σ^N ^operating under free-living growth conditions in *R. etli *[86]. In the *rpoN1 *mutant background, *prkA *showed an even stronger downregulation than in the *rsh *mutant during stationary phase, showing *prkA *transcription to be strongly σ^N^-dependent. Because NtrC is a common activator of σ^N^-dependent genes, a similar downregulation of *prkA *in the *ntrC *mutant was expected. However, no downregulation of *prkA *was observed during growth and early stationary phase in the *ntrC *mutant. Instead, *prkA *was highly upregulated during late stationary phase compared to the wild type, suggesting that *prkA *is under negative control of NtrC.

To further analyze the role of PrkA in cellular metabolism, we compared growth of the wild type and *prkA *mutant on 384 different nitrogen sources using glucose as the sole carbon source. Even though transcriptional control of *prkA *expression by *rpoN1 *suggests an involvement for PrkA in nitrogen metabolism, no growth defects were detected. Therefore, the specific function of this highly conserved protein in the (p)ppGpp regulon remains to be identified.

## Conclusions

**Figure 9:**
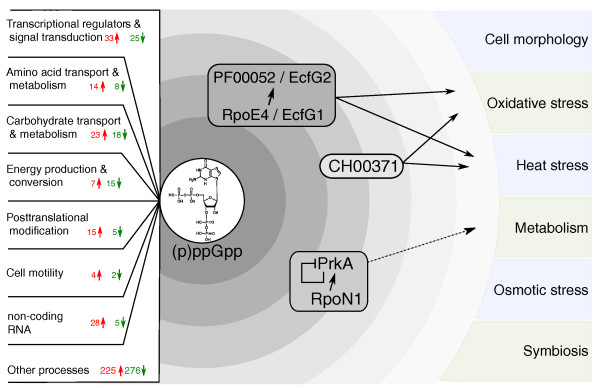
**The (p)ppGpp regulon of *R. etli***. The extensive impact of (p)ppGpp on gene expression of *R. etli *is illustrated by the number of up- and downregulated genes grouped according to functional categories. The remaining categories are combined as 'Other processes'. The *rsh *mutant is unable to synthesize (p)ppGpp and has a pleiotropic phenotype, such as an altered morphology, increased stress sensitivity and impaired symbiosis. As a global regulator, the regulon of (p)ppGpp is multilayered. Further insight into the (p)ppGpp-dependent stress response was obtained by the identification and subsequent characterization of three different regulators that are under strong positive regulation of (p)ppGpp during stationary phase. EcfG2/PF00052 and RpoE4, both ECF sigma factors, are partly functionally redundant for survival under heat stress and oxidative stress. The transcription factor CH00371 is also involved in survival during both heat and oxidative stress. PrkA, a serine kinase, likely plays a role in the (p)ppGpp-dependent adaptation of the cellular metabolism. Its transcription is positively controlled by RpoN1 and negatively autoregulated.

Analysis of growth phase-specific gene expression of the *R. etli *wild type and *rsh *mutant has provided insight into the (p)ppGpp regulon of *R. etli*, providing the first genome-wide view of the stringent response in an α-proteobacterium. Our results indicate that (p)ppGpp functions as a global regulator, with primarily an inducing role, in the adaptation to a non-growing lifestyle as shown by the extensive differential expression of genes belonging to all functional categories. Moreover, we showed both similarities and differences to its role in *E. coli *and other bacteria, reflecting the merit of investigating a well-studied regulatory response in species more distantly related to typical model organisms. Surprisingly, even though (p)ppGpp is considered to be a growth-arrest specific messenger, we identified a significant number of (p)ppGpp-dependent genes during early exponential phase as well, suggesting functional relevance of the low basal level of (p)ppGpp during active growth in *R. etli*. Additionally, the genome-wide transcriptome analysis of a strain deficient in a global regulator, and exhibiting a pleiotropic phenotype, enabled us to identify diverse regulators that control genes associated with a subset of stress phenotypes. The phenotypic analysis of three novel downstream regulators during stationary phase, that is, *ecfG2*, CH00371, and *prkA*, allowed us to obtain additional insight into the intricate regulatory role of this stress alarmone (Figure 9). Added detail to the complex picture of (p)ppGpp-dependent regulation of gene expression in *R. etli *was further provided by identifying several up- and downstream elements in the signal transduction cascades of these regulators. We conclude that (p)ppGpp is situated high up in the hierarchy of cellular gene regulation of *R. etli*, orchestrating its adaptation to growth stage or extracellular conditions through specific downstream regulators to control expression of a variety of target genes.

## Materials and methods

### Bacterial strains and growth conditions

The bacterial strains and plasmids used for this work are listed in Additional file 7. *R. etli *CFN42 strains were cultured in minimal AMS or complex TY medium at 30°C when used for RNA isolation or stress tests, respectively [29,87]. AMS medium was supplemented with 10 mM NH_4_Cl and 10 mM succinate unless otherwise indicated. *E. coli *strains were grown at 37°C in LB medium. In order to study gene expression during different growth phases in AMS medium, samples were taken based on optical density (OD) readings of OD_600 _= 0.3, OD_600 _= 0.7, and 6 hours after reaching the maximum optical density (OD)_600_, representing early exponential, late exponential and stationary phase, respectively [32]. Antibiotics were supplied at the following final concentrations (in μg^-1 ^ml): ampicillin, 100; gentamicin, 50; kanamycin, 25; spectinomycin, 50; nalidixic acid, 15; neomycin, 60; and tetracycline, 1 (*E. coli*) or 0.1 (*R. etli*).

### Mutant construction

The *R. etli *CFN42 *rsh *mutant was constructed by insertion of an Sp^R ^cassette, obtained from pHP45ΩSp, as described previously for *R. etli *CNPAF512 [29]. The *ecfG2 *and CH00371 mutants were constructed by first amplifying a 2.5-kb and a 1.9-kb fragment, respectively, using *Pfx *DNA polymerase and primers (CACC*GCGGCCGC*GGGTTT AAGGGGATAAATT and ACTG*GCGGCCGC*AAG GGCCGATCGAGATCCAC in the case of *ecfG2*; CACC*GCGGCCGC*AGCTGC AGGATCTTATGGGAATA and ACTG*GCGGCCGC*CGACGACCAGATCCTGAT CGC in the case of CH00371) that carried NotI recognition sites at their 5' ends (shown in italics). These fragments were subsequently cloned into pUC18Not, and a Km^R ^cassette flanked by transcription termination signals, obtained from pHP45ΩKm, was inserted in the HindIII and EcoRV site of CH00371 and *ecfG2*, respectively. From these plasmids, the corresponding NotI fragments were cloned into the suicide plasmid pJQ200uc1. For construction of the *rpoE4*-*ecfG2 *double mutant, an *ecfG2*::ΩSp suicide construct was obtained as described for the *ecfG2 *mutant above, replacing the Km^R ^cassette with a Sp^R ^cassette.

The non-polar *prkA *mutant was constructed by amplifying a 3.6-kb fragment using *Pfx *DNA polymerase and primers (CACC*GTTAAC*TCGACAGGAAAAGGTAG AGC and CACC*GTTAAC*TACTCG TCAAGAAGGAGGCT) that carried HpaI recognition sites at their 5' ends (shown in italics). This fragment was cloned into pCR4Blunt-TOPO (Invitrogen, Carlsbad, CA, USA). A fragment of 1.2 kb was removed from *prkA *by digesting with BamHI and XhoI (Figure 8a) and the construct was ligated after blunting, creating a deletion in *prkA*. An HpaI fragment was removed form this construct and cloned into the SmaI site of pJQ200uc1.

Finally, these suicide constructs were used for site-directed mutagenesis of the respective genes following triparental conjugation as described previously [88]. The obtained mutants were verified by Southern blot hybridization.

### RNA isolation and cDNA synthesis for microarray detection

RNA was isolated as described previously [32]. Briefly, the RNA content of bacterial cultures was stabilized using a phenol:ethanol solution. Pellets were frozen in liquid nitrogen and stored at -80°C. Total RNA was extracted using the TRIzol Plus RNA Purification kit (Invitrogen). DNA contamination was removed by TURBO DNase (Ambion, Austin, TX, USA)) and afterwards checked by PCR (45 cycles). To increase RNA yields and account for experimental variation, RNA from six different cultures was pooled. RNA integrity was analyzed using Experion RNA StdSens Chips (Biorad, Hercules, CA, USA) before and after precipitation. All samples had an RNA Quality Indicator value of 10. RNA quantity and purity was assessed using the NanoDrop ND-1000. The A260/A280 ratio and A260/A230 ratio of all samples were ≥2.

cDNA was synthesized using random decamers (Ambion) and the SuperScript Double-Stranded cDNA Synthesis Kit (Invitrogen) according to the manufacturer's protocol.

### High-density microarray design and data preprocessing

A whole-genome tiling array covering the entire *R. etli *genome sequence was used (see GEO GPL9409) and the data were analyzed as described previously [32]. Samples were hybridized and scanned by NimbleGen. The data were deposited in the NCBI Gene Expression Omnibus (GEO) and can be accessed through accession numbers [GEO:GSE23961], [GEO:GSM462173], [GEO:GSM462178], [GEO:GSM462180], [GEO:GSM590285], [GEO:GSM590286] and [GEO:GSM590287].

Differentially expressed genes were identified based on a standard deviation cutoff. These genes were considered induced or repressed if the absolute expression ratio was ≥2 (log_2 _≥1). This threshold is cogent since most regulatory responses in nature appear to function using low level changes as a kind of energy saving solution [89]. Hierarchical clustering was performed using the software package R.

### RT-(q)PCR

Expression levels were determined by RT-qPCR using SYBR Green, as described previously [32]. In short, primers were designed using Primer Express 3.0. Pooled total RNA (2 μg) of each growth condition (early/late exponential phase, stationary phase) was reverse transcribed to single-stranded cDNA using the SuperScript VILO cDNA Synthesis Kit according to the manufacturer's instructions (Invitrogen). DNA contamination of the RNA samples was checked by PCR (45 cycles) before RT. cDNA (40 ng) was used in each reaction. All reactions were performed in triplicate.

The microarray data were validated by determining the expression levels of 14 representative genes: *flaCh1*, *potF*, *rpsH*, *flgB*, *rplR*, *otsA*, *aglE*, a serine tRNA (CH01348), and genes encoding a chaperonin GroEL (CH00828), the sigma 54 modulation protein (CH00406), a permease protein of the Nod factor ABC transporter family (PD00277), the serine protein kinase *prkA*/CH02817, the transcriptional regulator CH00371 and the ECF sigma factor *ecfG2*/PF00052. The log_2 _ratios of the array data were compared to the log_2 _ratios of the qPCR results. 16S rRNA was not used as a reference gene as the level of mRNA/rRNA fluctuates during growth and the expression of rRNA is controlled by (p)ppGpp. New reference genes were identified using the geNorm algorithm in order to normalize the qPCR data [90]. Based on the microarray data, five genes were chosen that were relatively stable across all samples and assumed not to be co-regulated. These candidate reference genes were *greA*, *cinRa*, *tatA*, and genes encoding a zinc binding protein (CH00586) and a hypothetical protein (CH01579). 16S rRNA was included for comparison. Using geNorm, we determined *repBa2 *and *tatA *to be the most stable reference genes as they have the lowest gene expression stability values M (Figure S3a in Additional file 8). Consequently, a gene expression normalization factor could be calculated for each sample using the most stable genes. By plotting the pairwise variation V between two sequential normalization factors containing an increasing number of genes, we determined that the three best reference genes were an optimal number of reference genes for normalization (Figure S3b in Additional file 8). Although V_2/3_, the pairwise variation between the normalization factors calculated by the two and three most stable genes, is strictly higher then the proposed 0.15 cutoff value of Vandesompele *et al*. [90], the difference is very small and the pairwise variation decreases only slightly by taking an additional fourth reference gene. Therefore, the normalization factor would not significantly change if more internal control genes were to be included. Also, the degree of resolution does not require a fourth reference gene.

RT-PCR was performed on cDNA samples (40 ng) of stationary phase to determine the operon organization of *prkA*. The primers were designed accordingly (Additional file 6). *Taq *DNA polymerase was used in the PCR reactions (35 cycles).

### Construction of *prkA*-*gusA *promoter fusion and β-glucuronidase assay

The *prkA*-*gusA *reporter fusion was constructed by first amplifying the 400 bp upstream of *prkA *by PCR using *Pfx *DNA polymerase and primers (ACTG *AAGCTT*TCTGCGGTTCGCCTATCGCA and ACTG*TCTAGA*AGCGCCGGAAG CGTATGATC) that carried a HindIII and XbaI recognition site at their 5' end (shown in italics), respectively. This promoter fragment was cloned into pFAJ1703 after digestion with HindIII and XbaI, thereby flanking the promoterless 5' end of *gusA*. Quantitative analysis of GusA activity was carried out as described previously [29].

### Stress and stationary phase survival

To study stress survival, wild-type and mutant cells from a freshly grown culture on a MM79 agar plate were resuspended in 10 mM MgSO_4 _at an OD_600 _of approximately 0.5. For each regulator, two independently constructed mutants were analyzed in order to exclude the involvement of secondary mutations. To test heat stress survival, 1 ml of each sample was incubated at 45°C for 30 minutes. In case of oxidative stress, 0.1 ml of 100 mM H_2_O_2 _was added to 0.9 ml of each sample for 30 minutes or 1 hour while for osmotic stress 0.5 ml of 5 M NaCl was added to 0.5 ml of sample. Samples were plated on TY agar containing nalidixic acid using the Eddy Jet spiral plater (IUL Instruments, Barcelona, Spain). Control samples were incubated without the stress agent at 30°C and the colony forming units (CFU) were determined at the same time point as the stressed samples. The total number of CFU per ml was determined after 3 days of incubation at 30°C using the Flash and Go automated colony counter (IUL Instruments). All experiments were repeated at least two times using three independent biological replicates.

To assess long-term survival, pellets of overnight cultures of wild-type and mutant strains were washed and resuspended in 10 mM MgSO_4 _at an OD_600 _of 0.5. A volume of 100 ml of AMS medium (10 mM NH_4_Cl and succinate) was inoculated with 1 ml of cell suspension and incubated at 30°C for 2 weeks. Samples of 1 ml were removed at the indicated time points and subjected to viable cell counts as described above.

### Swimming test

To study swimming activity, TY plates containing 0.2% agar were spot inoculated with cultures in exponential phase and incubated at 30°C in a closed container as described previously [91]. Each strain was tested five-fold in two independent experiments. The swimming halo diameter was measured after one, two and three days.

### Growth analysis

Biolog Phenotype Microarray panels PM3/6/7/8 were used to test growth on nitrogen sources and PM10 was used to test pH susceptibility (Biolog, Hayward, CA, USA). AMS medium was inoculated (1:1,000) with overnight cultures of *R. etli *strains, washed and the OD_600 _was corrected to approximately 0.5. No NH_4_Cl was added in case of PM3/6/7/8. The Biolog redox indicator dye Mix A was added to the medium (1:100). The microplates were loaded with 100 μl in each well and incubated for 7 days at 30°C. Dye reduction was monitored every 12 h by measuring the OD_570 _using a Synergy Mx Microplate Reader (BioTek, Winooski, VT, USA).

## Abbreviations

CFU: colony forming units; ECF: extracytoplasmic function; IS: insertion sequence; ncRNA: non-coding RNA; OD: optical density; ppGpp: guanosine tetraphosphate; pppGpp: guanosine pentaphosphate; RNAP: RNA polymerase; *rrn*: ribosomal RNA; RT-qPCR: reverse transcription-quantitative polymerase chain reaction.

## Authors' contributions

MV carried out the experiments and bioinformatics analysis. MV, MF, KB, and JM conceived the study and contributed to the interpretation of the data. LC, KE, and KM performed and contributed to the microarray data normalization and processing. MV, MF and JM were involved in drafting the manuscript. All authors read and approved the final manuscript.

## Supplementary Material

Additional file 1 **Figure S1. Growth curve of *R. etli *CFN42 in AMS medium**. (a) Optical density (OD) readings during growth of the wild type and *rsh *mutant shown in green and red, respectively. The arrows indicate the time points of sampling. (b) Colony forming units (CFU) during growth of the wild type and *rsh *mutant. Click here for file

Additional file 2 **Figure S2. MA plots comparing transcriptome data**. Scatter plots of the microarray data that plot the distribution of the log_2 _intensity ratio (M-value) versus the log_2 _average intensity (A-value). Differentially expressed genes that are upregulated or downregulated are shown in red or green, respectively. The number of genes with a growth phase or (p)ppGpp-dependent expression profile are indicated by histogram bars at the right of the MA plot. **(a) **Wild type compared to *rsh *mutant in stationary phase. **(b) **Wild type compared to *rsh *mutant in exponential phase. Click here for file

Additional file 3 **Table S1**. The differentially expressed genes during stationary phase and exponential phase in the wild type compared to the *rsh *mutant. Click here for file

Additional file 4 **Table S2**. The RpoE4-regulated genes according to Martinez-Salazar *et al*. (2009) that were found to be alarmone-dependent in this study [42]. Click here for file

Additional file 5 **Table S3**. The alarmone-dependent ncRNAs. Click here for file

Additional file 6 **Table S4**. The RT-qPCR fold changes compared to array fold changes and qPCR primers. Click here for file

Additional file 7 **Table S5**. The bacterial strains and plasmids used in this study. Click here for file

Additional file 8 **Figure S3**. RT-qPCR identification of stable endogenous genes. **(a) **Determining the most stable reference genes using the average expression stability value M of the remaining reference genes during a stepwise exclusion of the least stable internal control gene. The genes are ranked according to increasing expression stability. At the left are the least stable genes and at the right are the most stable ones. **(b) **Determining the optimal number of reference genes using the pairwise variation V between two sequential normalization factors containing an increasing number of genes with 0.15 as a proposed cutoff value by Vandesompele *et al*. [90]. Click here for file
