# Supplementary figures and images for: Stress response regulators identified through genome-wide transcriptome analysis of the (p)ppGpp-dependent response in Rhizobium etli
Source: Genome Biol. 2011 Feb 16;12(2):R17. doi: 10.1186/gb-2011-12-2-r17 (PMC3188799; doi:10.1186/gb-2011-12-2-r17)

**A**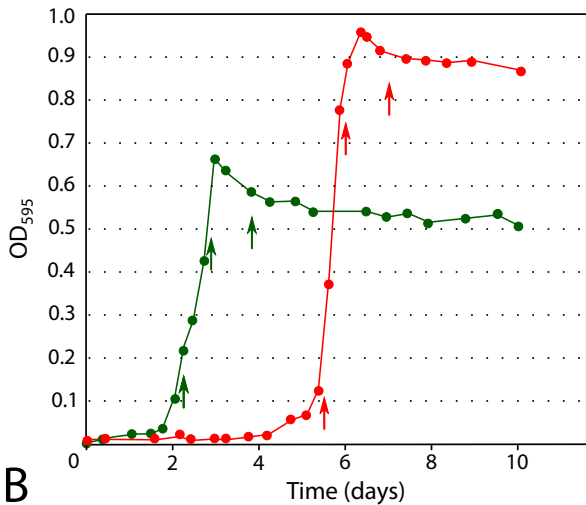**B**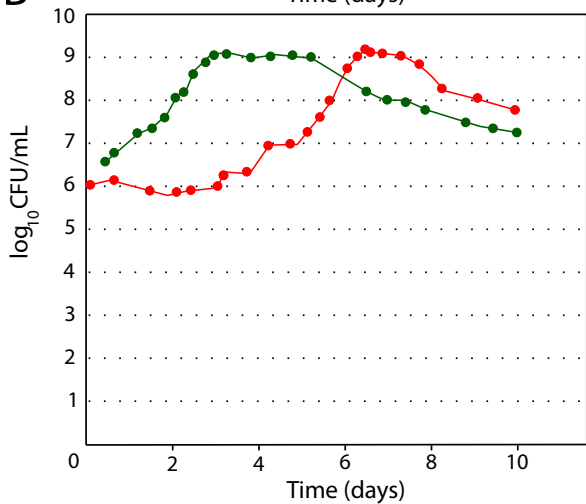

Supplement: Additional file 1 — Figure S1. Growth curve of R. etli CFN42 in AMS medium. (a) Optical density (OD) readings during growth of the wild type and rsh mutant shown in green and red, respectively. The arrows indicate the time points of sampling. (b) Colony forming units (CFU) during growth of the wild type and rsh mutant. [file gb-2011-12-2-r17-S1.PDF]

**Wild type vs *rsh* mutant stationary phase**

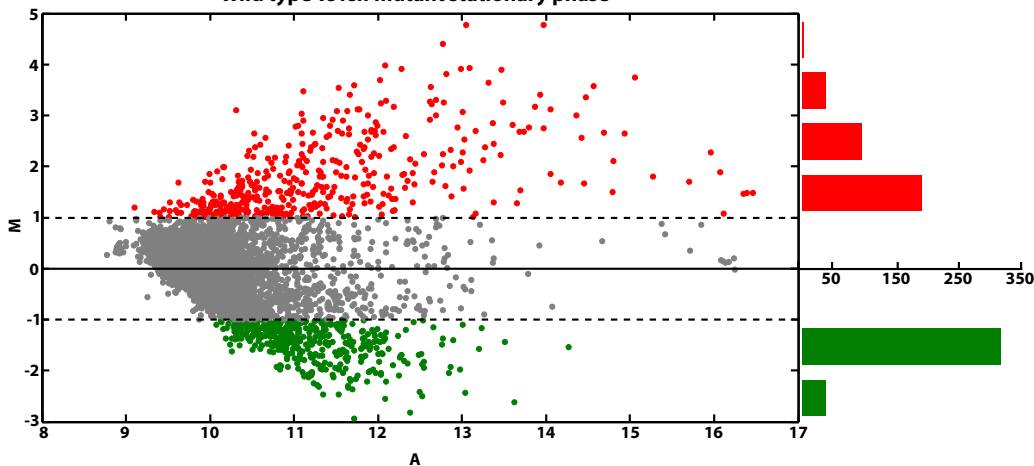

**Wild type vs *rsh* mutant early exponential phase**

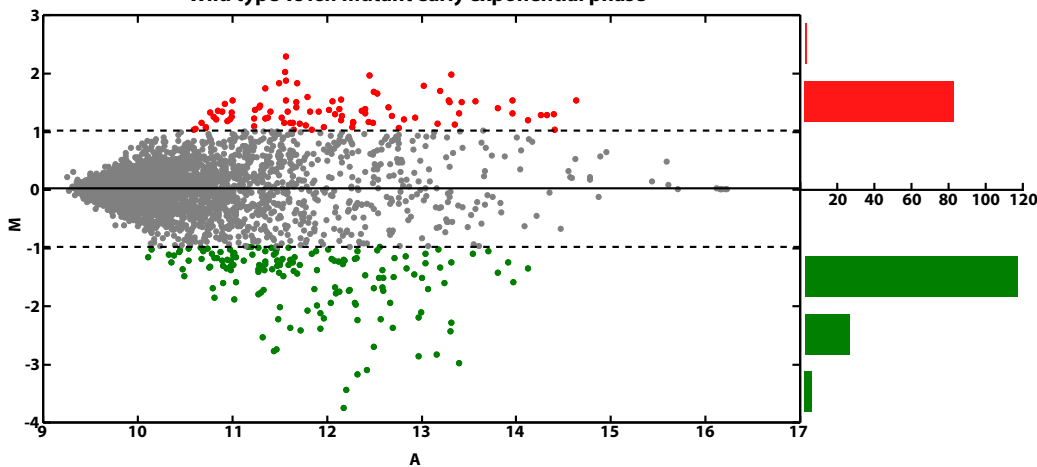

Supplement: Additional file 2 — Figure S2. MA plots comparing transcriptome data. Scatter plots of the microarray data that plot the distribution of the log2 intensity ratio (M-value) versus the log2 average intensity (A-value). Differentially expressed genes that are upregulated or downregulated are shown in red or green, respectively. The number of genes with a growth phase or (p)ppGpp-dependent expression profile are indicated by histogram bars at the right of the MA plot. (a) Wild type compared to rsh mutant in stationary phase. (b) Wild type compared to rsh mutant in exponential phase. [file gb-2011-12-2-r17-S2.PDF]

**A**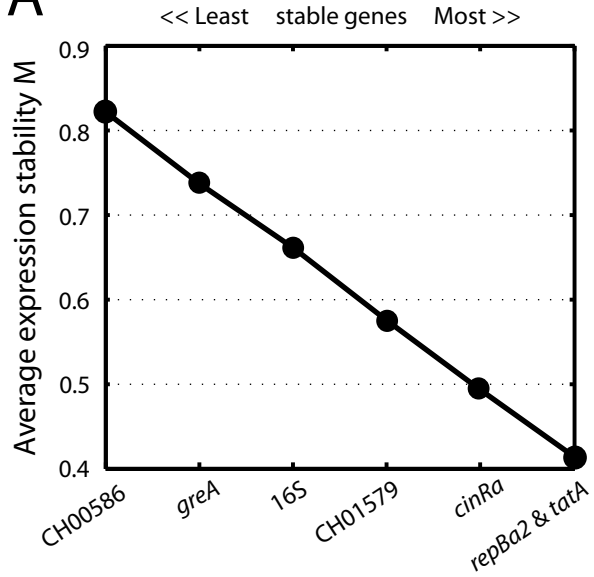**B**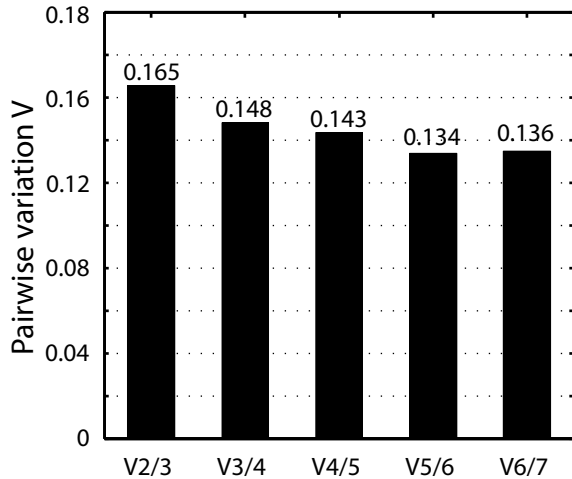

Supplement: Additional file 8 — Figure S3. RT-qPCR identification of stable endogenous genes. (a) Determining the most stable reference genes using the average expression stability value M of the remaining reference genes during a stepwise exclusion of the least stable internal control gene. The genes are ranked according to increasing expression stability. At the left are the least stable genes and at the right are the most stable ones. (b) Determining the optimal number of reference genes using the pairwise variation V between two sequential normalization factors containing an increasing number of genes with 0.15 as a proposed cutoff value by Vandesompele et al. [90]. [file gb-2011-12-2-r17-S8.PDF]
